# A Hybrid Feature Selection and Multi-Label Driven Intelligent Fault Diagnosis Method for Gearbox

**DOI:** 10.3390/s23104792

**Published:** 2023-05-16

**Authors:** Di Liu, Xiangfeng Zhang, Zhiyu Zhang, Hong Jiang

**Affiliations:** College of Intelligent Manufacturing and Industrial Modernization, Xinjiang University, Urumchi 830017, China

**Keywords:** fault diagnosis, compound fault decoupling, gearbox, feature selection, multi-label learning

## Abstract

Gearboxes are utilized in practically all complicated machinery equipment because they have great transmission accuracy and load capacities, so their failure frequently results in significant financial losses. The classification of high-dimensional data remains a difficult topic despite the fact that numerous data-driven intelligent diagnosis approaches have been suggested and employed for compound fault diagnosis in recent years with successful outcomes. In order to achieve the best diagnostic performance as the ultimate objective, a feature selection and fault decoupling framework is proposed in this paper. That is based on multi-label K-nearest neighbors (ML-kNN) as classifiers and can automatically determine the optimal subset from the original high-dimensional feature set. The proposed feature selection method is a hybrid framework that can be divided into three stages. The Fisher score, information gain, and Pearson’s correlation coefficient are three filter models that are used in the first stage to pre-rank candidate features. In the second stage, a weighting scheme based on the weighted average method is proposed to fuse the pre-ranking results obtained in the first stage and optimize the weights using a genetic algorithm to re-rank the features. The optimal subset is automatically and iteratively found in the third stage using three heuristic strategies, including binary search, sequential forward search, and sequential backward search. The method takes into account the consideration of feature irrelevance, redundancy and inter-feature interaction in the selection process, and the selected optimal subsets have better diagnostic performance. In two gearbox compound fault datasets, ML-kNN performs exceptionally well using the optimal subset with subset accuracy of 96.22% and 100%. The experimental findings demonstrate the effectiveness of the proposed method in predicting various labels for compound fault samples to identify and decouple compound faults. The proposed method performs better in terms of classification accuracy and optimal subset dimensionality when compared to other existing methods.

## 1. Introduction

Gearboxes are widely used as important power transmission components in industrial systems; however, due to the harsh working environment and the combined effect of multiple sources of excitation, gearbox failures are inevitable. In the actual environment, a failure often does not occur alone but in the same component or different components at the same time as multiple independent single failures, which constitutes a compound failure. For the whole mechanical system, the destructiveness of compound faults is much greater than that of single faults [1], so it is important to make an accurate diagnosis of compound faults in gearboxes. Moreover, when one component of a gearbox fails, it affects the vibration characteristics of another component [2], which makes it possible that considering only a single component may lead to erroneous results, so it is extremely important to monitor the operating conditions of each component in the gearbox simultaneously.

Compound faults have made fault diagnosis and predictive maintenance of mechanical equipment dramatically more difficult, and decoupling compound failures remains a major challenge today [3]. In recent years, data-driven approaches have provided a promising approach for condition monitoring and fault diagnosis, where various data collected from the system are used to train artificial intelligence-based predictive models [4]. Since vibration signals are most sensitive to changes in the implemented operating state of the equipment, and the signal features have a strong correspondence with the fault state of each component inside the equipment, they are often used as a carrier of fault information [5]. As the equipment structure becomes more complex and the phenomenon of coupling multiple fault information becomes more tricky, the requirements for diagnosing faults are getting higher and higher, and obtaining higher recognition accuracy and reducing the training cost of the model are constantly pursued goals. In data-driven fault diagnosis methods, the selection of the most representative features is crucial to the classification performance of the prediction model, so we intend to propose a method that automatically selects the optimal subset optimized for the classifier from the original high-dimensional feature set, with the ultimate goal of achieving decoupling of compound faults and obtaining the highest recognition accuracy.

## 2. Motivation and Literature Review

As mentioned in the previous section, data-driven intelligent fault diagnosis methods have considerable potential, especially the introduction of computer theoretical technical disciplines such as machine learning (ML) and deep learning (DL) has had a rather positive impact, and many studies have shown the effectiveness of using them as classifiers [6,7]. However, the lack of interpretability, high computational cost and high dependence on tuning parameters make DL less practical and reliable than ML in most scenarios [8]. Several ML-based fault classification models [9,10,11] have demonstrated the effectiveness of these techniques in fault diagnosis.

Data-driven fault diagnosis algorithms are usually composed of three parts: feature extraction, feature selection, and fault classification [12]. Among them, the set of fault features constructed during feature extraction is crucial to achieving an accurate diagnosis. Since the collected vibration signals are often a mixture of signals from different components, this leads to the fact that the fault type will become difficult to diagnose when different components fail at the same time, influenced by the coupling of fault features with each other [13]. Coupled with the complexity of the compound fault mechanism, it is difficult for a single feature or single-domain feature to accurately describe the changes in the operating state of the equipment, while the construction of a multi-domain feature set can comprehensively characterize the changing trend of the equipment fault state from multiple feature spaces. Some existing research works have shown that multi-domain feature sets have better performance in fault diagnosis compared to constructing single-domain feature sets [14]. Therefore, in order to effectively characterize the gearbox operating state, it has become a common practice to extract a large number of representative features, such as time domain and frequency domain features from the signal, and then construct a feature set that contains as much fault information as possible, which is widely used by researchers in the field of fault diagnosis.

Although constructing a multi-domain feature set that is as comprehensive as possible can effectively overcome these problems, it will also inevitably lead to a feature set that will contain some redundant features or irrelevant features [15], which may reduce computational efficiency and diagnostic accuracy. Additionally, a fault feature set with an excessive amount of dimensionality will also pose a severe test for the subsequent steps. Therefore, dimensionality reduction techniques are used to obtain an optimal subset from the original feature set with moderate dimensionality and eliminate irrelevant and redundant features. There are two major types of mainstream dimensionality reduction techniques that are often applied in fault feature selection. One of them is called feature reduction, which maps the feature set from a high-dimensional feature space to a low-dimensional feature space and reduces the dimensionality by constructing new features; the typical methods are principal component analysis (PCA) [16], linear discriminant analysis (LDA) [17], etc.

However, the physical meaning of new features generated by feature reduction methods is far from the original features, blurring the information of the original features or even the opposite. In contrast, feature selection can retain the original physical meaning of fault features by selecting the most characterized sensitive features from the original high-dimensional feature set without changing the original feature structure [18], which has good interpretability, and this physical meaning can be used to analyze the formation mechanism of faults and the design of suitable classifiers [19]. Feature selection algorithms can be classified as Filter, Wrapper, and Hybrid based on the degree of dependence on the classifier in the selection process [20,21,22]. The Filter algorithm, which is separate from the classifier and is typically used as a preprocessing step to provide the classifier with a reduced feature set, can quickly eliminate a large number of irrelevant features from the original set [23,24]. The Wrapper algorithm combines with the classifier in the selection process, uses the classification accuracy as the evaluation criterion to measure the quality of the feature subset, and the final retained feature subset has very good classification performance [9,25]. In fact, any search strategy can be used as a guide to the search direction of the Wrapper algorithm, so the search process has a strong generalization capability, but it also results in an extremely high time complexity.

As mentioned above, the benefits and drawbacks of the Filter and Wrapper algorithms appear to be complementary. Therefore it is an effective strategy to use the ranking information obtained by the Filter algorithm to guide the search direction of the Wrapper method [26] and balance the classification accuracy and computational cost; most of the existing hybrid feature selection algorithms are designed as a two-stage framework. Zhang et al. [5] and Liang et al. [27] first used the Filter method to obtain a pre-selected subset and then further searched for optimal features using a genetic algorithm and particle swarm optimization, respectively. Mochammad et al. [28] first selected five features from each of the original feature sets using three different Filter models and then searched for the optimal subset by exhausting the combinations among these features. Ganjie et al. [29] first ranked the features according to their relevance to class labels, then applied different clustering methods to divide them into multiple subsets, and finally obtained the optimal subset by traversing all subsets. Chen et al. [11] applied the evolutionary multitasking technique for feature selection by estimating weights for features by Relief-F in the first stage and dividing the feature set into two tasks based on the weights. In the second stage, the optimal subset is obtained by knowledge transfer between two tasks and using particle swarm optimization.

The overall objective of this paper is to develop an efficient feature selection and fault decoupling framework. In the original feature extraction stage of the proposed intelligent diagnosis method, the effect of noise is diminished through pre-processing, and the original high-dimensional feature set is constructed by extracting statistical features in the time domain and frequency domain. Then, the Filter model is used for feature pre-ranking, the weighting scheme is used for feature re-ranking, and the Wrapper model is used for correctly pinpointing the optimal subset automatically in the proposed three-stage feature selection framework. Finally, the decoupling of compound faults is accomplished using ML-kNN as a classifier. Specifically, this study contributes the following:

- A feature set construction method that efficiently handles a low signal-to-noise ratio and nonlinear signals using statistical feature extraction techniques;
- A feature selection framework that is applicable to multi-label data and can automatically search for the optimal subset from the original high-dimensional feature set;
- A physical interpretative fault diagnosis framework that can maximize the preservation of fault features.

The remainder of this paper is organized as follows. The theoretical basis of the main components of the proposed method is briefly covered in Section 3. Section 4 provides a detailed description of the proposed method. In Section 5, the method is applied to real data examples of bearing-gear compound fault detection and compared with some classical methods. Finally, Section 6 draws conclusions and discusses possible directions for future work.

## 3. Theoretical Basis of the Study

This section introduces the theoretical background of the feature selection model and multi-label learning technology used in the research.

### 3.1. Feature Selection Models

#### 3.1.1. Filter Models

In the Filter method, commonly used evaluation criteria include distance measure, information measure and correlation measure. For example, the Fischer score method emphasizes the distance between labels and is based on distance measurement. The information gain method assesses features from the viewpoint of information and is based on information measures. Based on correlation measures, the Pearson correlation coefficient method emphasizes the correlation between feature and label.

- Fischer score:

In the Fisher score (FS), each feature is selected independently according to its Fisher criterion score [30], and the aim is to find features that satisfy the distance that maximizes the inter-class distance and minimizes the intra-class distance. Assume that there are *n* samples in the feature set belonging to *Y* labels, each of which contains *n_k_* samples. Define the inter-lass scatter *S_b_*(*f_i_*) of the *i*th feature *f_i_* as:

(1) $$S_{b}(f_{i})=\sum_{k=1}^{Y}n_{k}{\left(m_{i}{}^{k}-m^{k}\right)}^{2}$$

where *n_k_* represents the number of samples in the *k*th category, *m^k^_i_* represents the mean of the values taken by the samples in the *k*th category on the *i*th feature, and *m_k_* represents the mean of the values taken by the samples in all categories on the *i*th feature.

Define the intra-class scatter *S^k^_t_*(*f_i_*) of the *k*th sample at the *i*th feature *f_i_* as:

(2) $$S_{t}^{k}(f_{i})=\sum_{j=1}^{n_{k}}{\left(x_{i,j}^{k}-m_{i}^{k}\right)}^{2}$$

where *x^k^_i_*_,*j*_ is the value of the *j*th sample that belongs to the *k*th class of samples in the *i*th feature.

When the inter-class scatter *S_b_*(*f_i_*) of the *i*th feature is larger, and the intra-class scatter *S^k^_t_*(*f_i_*) is smaller, the stronger the characterization ability of the feature is, and the calculation formula for FS can be obtained as follows:

(3) $$Fs(f_{i})=\frac{S_{b}(f_{i})}{\sum_{k=1}^{C}n_{k}S_{t}^{k}(f_{i})}=\frac{\sum_{k=1}^{C}n_{k}{\left(m_{i}^{k}-m^{k}\right)}^{2}}{\sum_{k=1}^{C}\left[n_{k}\sum_{j=1}^{n_{k}}{\left(x_{i,j}^{k}-m_{i}^{k}\right)}^{2}\right]}$$

Following FS evaluation, the smaller the obtained score, the less significant the features are, whereas the larger the obtained score, the higher the feature importance and the stronger the characterization ability. However, the Fisher score does not measure the correlation degree between features, so it cannot eliminate the redundant features in the original set;

- Information gain:

In the 1940s, Shannon first introduced the concept of information entropy and provided a mathematical expression to calculate it [31], which allowed the value of information to be expressed quantitatively and provided a standard by which the value of information could be determined. For a label *Y* = {*y*_1_, *y*_2_, ⋯, *y_n_*} in a classification task, and assuming that the probability corresponding to the value taken in the label is *P*(*y_i_*), the information entropy that this label has is given by the following equation:

(4) $$H(Y)=-\sum_{i}^{n}P(y_{i})\log_{2}(P(y_{i}))$$

From Equation (4), it can be seen that the greater the uncertainty of the label *Y*, the greater the entropy, implying that more information is needed to determine the label as well. Assuming that a feature in the feature set can be represented as *X* = {*x*_1_, *x*_2_, ⋯, *x_n_*}, the conditional entropy that *X* has is given by the following equation:

(5) $$H(Y|X)=-\sum_{j=1}^{n}P(g_{j})\sum_{i=1}^{l}P(y_{i}|x_{i})\log_{2}(P(y_{i}|x_{i})$$

*P*(*y_i_*) in Equation (4) represents the prior probability of label *Y*, and *P*(*y_i_*|*x_i_*) in Equation (5) represents the conditional probability of label *Y* when feature *X* is determined. Information gain (IG) is a measure of the difference between the prior uncertainty of a feature and the expected posterior uncertainty, and for classification tasks, information gain is calculated by first calculating the feature’s occurrence in each class number of occurrences of the feature in each class, which is then used to describe the information gain of the feature for each class [32]. Given the feature *x* and its corresponding label *y*, the IG is calculated as follows:

(6) I(x;y)=H(y)−H(y|x)

From the definition, when *H*(*y*) − *H*(*y*|*x*) = 0, it means that the label *y* is completely irrelevant to the feature *x*, while when *I*(*x*; *y*) > 0, it means that the label *y* is related to the feature *x*. And the larger *I*(*x*; *y*) is, the closer the association between the label and the feature; that is, the greater the information gain of the feature, the better its characterization ability and the more critical for fault classification;

- Pearson correlation coefficient:

Pearson correlation coefficient (PCC) is used to measure the degree of strength of linear correlation between variables [33] and can also be used to measure the correlation between features and labels. A feature with excellent representational power has a strong correlation with the label, while the features should be uncorrelated with each other. The PCC between two variables is defined as the quotient of their covariance and standard deviation:

(7) $$\rho (x,y)=\frac{\mathrm{cov}(x,y)}{\sigma_{x}\sigma_{y}}=\frac{E[(x-\mu_{x})(y-\mu_{y})]}{\sigma_{x}\sigma_{y}}$$

Equation (7) is commonly used to predict continuous outcomes, and when generalized to the multi-classification case, given a feature *x* and its corresponding label *y*, the PCC is calculated as follows:

(8) $$\rho (x)=\frac{\sum_{i=1}^{m}\left(x_{i}-\bar{x})(y_{i}-\bar{y}\right)}{\sqrt{\sum_{i=1}^{m}{\left(x_{i}-\bar{x}\right)}^{2}\sum_{i=1}^{m}{\left(y_{i}-\bar{y}\right)}^{2}}}$$

Obviously, the larger the value of *ρ*, the stronger the correlation between features and labels, and the greater the gain that can be provided for the fault classification task.

#### 3.1.2. Wrapper Models

In general, there are three basic search strategies in the Wrapper method: global search strategy, sequential search strategy, and random search strategy [34,35]. The goal of the global search strategy is finding the optimal global subset of the original feature set, but this goal can only be guaranteed when almost all permutations have been exhausted, which is obviously extremely unrealistic for high-dimensional sets. Sequential search strategy and random search strategy are collectively referred to as heuristic search strategies, which add constraints to the search process to guide the search in the optimal direction. As a result, they have lower time complexity than the global search strategy, but the search results are usually inferior to global search. However, this sacrifice is worthwhile considering the amount of computation that can be saved by applying heuristic search strategies.

- Binary search:

Binary search (BS), also known as half search, starts from the middle element when searching and stops searching if the middle element meets the search requirement; otherwise, it continues from the middle element and searches until it finds the solution. The search process is as follows: the original feature set is split into two roughly equal parts, the left subset and the right subset. If the classification error rate of the left subset is below a threshold, the left subset is retained for further search and the right subset is deleted, and vice versa. Obviously, each search can narrow the search by half. Until the next search, the iterative process stops when the error rates of both the left and right subsets are higher than the set threshold.

BS is a very effective search strategy, but the prerequisite for it to give full play to its advantages is that the search object must be a set that conforms to the sequential structure. That is, each candidate element in the original set should be ordered according to some rules; otherwise, the search process will be meaningless;

- Sequential forward search:

Sequential forward search (SFS) is a bottom-up search strategy similar to the enumeration method. After selecting an optimal feature, which is the most beneficial one for the classification task, each feature selected thereafter is the feature that performs best when combined with the selected features. The specific search process of SFS is to add one feature at a time to the initial subset, and the iterative process stops when the classification accuracy does not improve with the increase of features;

- Sequential backward search:

Sequential backward search (SBS) is a top-down search strategy, and its search strategy is the opposite of SFS, which starts from all features and eliminates one feature at a time and can make the remaining features perform optimally when combined. The specific search process is to reduce one feature at a time in the initial subset, and the iterative process stops when the classification accuracy does not improve with the reduction of features.

### 3.2. Multi-Label Classification

Unlike traditional machine learning methods, the samples processed in the multi-label learning task contain multiple labels at the same time, which is more in line with the scenarios faced during real-world applications. More importantly, treating compound faults as an independent fault type means that a completely new label needs to be assigned to each compound fault state. The number of combinations formed will increase exponentially as the number of fault categories or the degree of fault changes, which makes it extremely impractical to obtain sufficient samples for each fault state, and the difficulty of fault classification will increase significantly. The number of labels required for compound fault diagnosis will be drastically reduced in multi-label learning, on the other hand, because the compound fault states will be simultaneously classified into multiple labels that correspond to a single fault during diagnosis and will no longer need additional labels.

Existing multi-label learning methods can be classified into two main categories: problem transformation (PT) and algorithm adaption (AA). In brief, PT algorithms make multi-label data adaptive to existing algorithms, while AA algorithms are adaptive to multi-label data [36]. In other words, the PT method tends to convert multi-label data into single-label data, which in turn converts the multi-label classification problem into multiple simple single-label classification problems. The AA method, on the other hand, tends to improve existing single-label classification algorithms so that these algorithms can directly deal with multi-label data.

The effectiveness of converting multi-label data into single-label data by the PT method and then performing feature selection has been demonstrated by a large number of researchers, such as Oscar et al. [37] used the PT method to convert multi-label problems into single-label problems and executed Relief-F algorithm on the single-label dataset converted by PT method. Newton et al. [38] combined two PT methods and two Filter molders to obtain four multi-label feature selection methods. Through numerous sets of experiments, they showed how effectively the single-label feature selection algorithm worked with the transformed dataset. Therefore, we intend to first convert the original multi-label data into single-label data by the PT method, then execute the feature selection method, and finally achieve the diagnosis and decoupling of compound faults by the AA classifier.

#### 3.2.1. Label Powerset

Label powerset (LP) [39] is a straightforward and effective PT method that converts an *L*-class multi-label problem into a multi-class single-label problem with 2^|*L*|^-class labels. Its processing can be briefly summarized as treating the different combinations of labels present in the training set as a new class label, thus converting the multi-label data into single-label data and taking the correlation between the labels into account during the conversion process [37,40].

#### 3.2.2. Multi-Label k-Nearest Neighbor Classifier

ML-kNN is a classical AA method [41], which is derived from the kNN algorithm. The fundamental procedure can be succinctly stated as follows: The k-nearest neighbor in the training set is initially determined for each test sample. The label set of the test instance is then determined using the maximum a posteriori probability principle based on the statistics obtained from the label set of these adjacent instances, that is, the number of adjacent instances belonging to each possible category. Since ML-kNN determines the k-nearest neighbor of the test sample through the distance between the test sample and all the samples in the training set and then determines its category, without a complicated mapping process, it is explainable with clear physical meaning.

## 4. The Proposed Methodology

### 4.1. An Overview of the Proposed Feature Selection and Fault Decoupling Method

**Figure 1:**
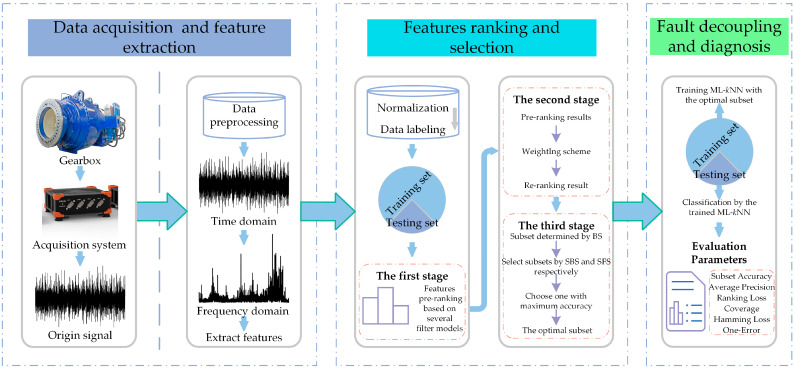
The flowchart of the proposed feature and fault decoupling method.

The flowchart of the proposed feature selection scheme and fault decoupling procedure is shown in Figure 1. The proposed feature selection and fault decoupling method includes data extraction and feature extraction, feature ranking and selection, and fault decoupling and diagnosis, as described below.

**Figure 2:**
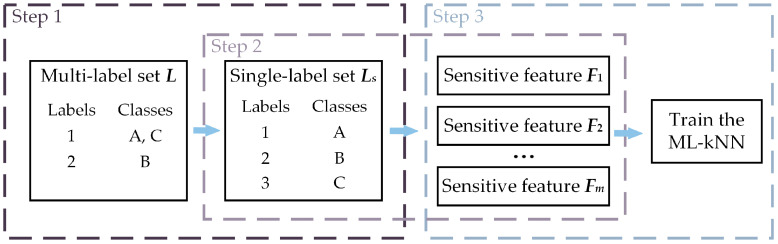
The feature selection process for multi-label data.

1. Data acquisition and feature extraction: A data acquisition system is used to obtain the gearbox’s vibration signal. In order to more effectively remove disturbing components from the signal and highlight fault characteristic information, the raw signal is first pre-processed and then time domain statistical features and frequency domain statistical features are extracted from the raw and pre-processed signals, as shown in Section 4.2;
2. Feature ranking and selection: In this paper, we will use the LP method to transform the multi-label data and perform feature selection before utilizing ML-kNN as a classifier to evaluate the effectiveness of feature selection. In other words, the PT method is merely utilized to achieve feature selection, and once feature selection for the original feature set has been finished, the original multi-label problem will be taken into account once more to achieve multi-label classification using the AA method. As a result, our proposed processing method consists of 3 primary steps, as shown in Figure 2. The LP method is used in step 1 to transform the multi-label set *L* into ingle-label set *Ls*. The proposed feature selection method, which is illustrated in Section 4.3, is used to search the most sensitive features from the origin feature set in step 2. In step 3, the quality of the optimal subset *Lm* is evaluated by ML-kNN.
3. Fault decoupling and diagnosis: Using the index of the optimal features obtained through the training set, the corresponding optimal features are selected from the original high-dimensional feature set of the testing set. Finally, the fault decoupling results of the testing set are obtained by the trained ML-kNN classifier, which is trained by the training set.

### 4.2. Original Feature Extraction of Vibration Signals

**Figure 3:**
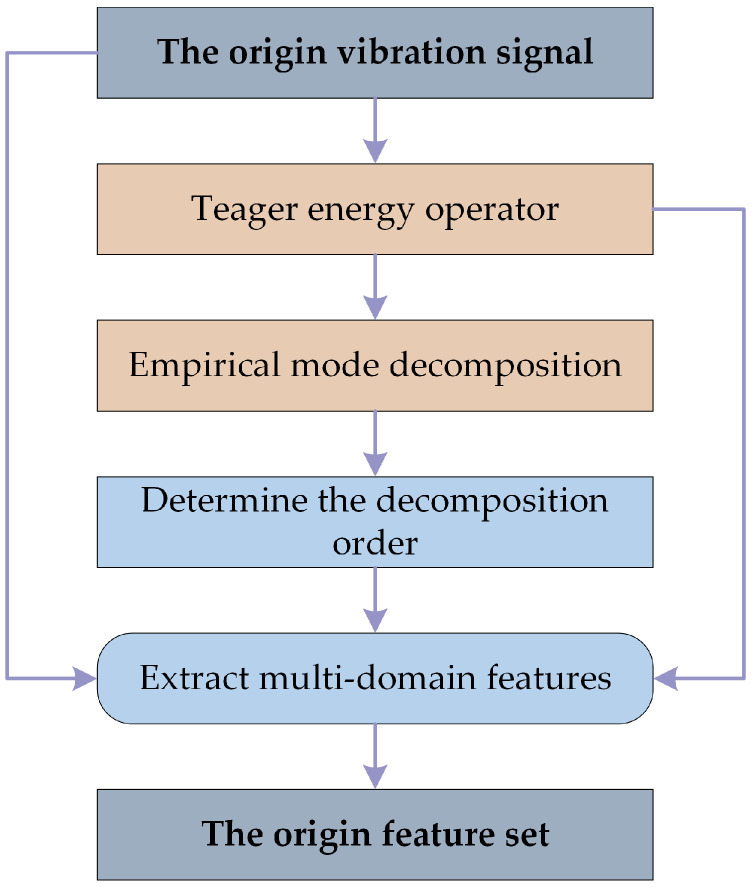
The flowchart of original feature extraction.

The obtained vibration signal is generally non-smooth and non-linear and has a low signal-to-noise ratio since it is restricted by the actual environment. This raises the difficulty of feature extraction and makes it more difficult to extract fault information from the vibration signal. It can often be difficult to adequately describe the fault state of the equipment by extracting only single or single domain features due to the complicated coupling behavior in the compound fault. Therefore, in this paper, the Teager energy operator and empirical modal decomposition (EMD) algorithm will be used to preprocess the signal and then extract the statistical features from the vibration signal, and the process is shown in Figure 3.

Specifically, to emphasize the transient components of the signal, the paper first demodulates the original signal using the Teager energy operator [42]. Then the vibration signal is divided into numerous intrinsic mode functions (IMFs) via the EMD algorithm. These IMFs contain local characteristic signals of the original signal at different time scales [43], and each component exhibits singularity and reflects a piece of unique frequency information in the signal. The first few orders of IMF that contain almost all the information are selected, and then time domain features and frequency domain features are extracted from them to construct multi-domain feature sets. These features are summarized in Table A1, denoted as T1–T11 and F1–F13, respectively, and more information on their physical significance can be obtained in the literature [44]. In order to obtain more fault information, features will be extracted from both the original and demodulated signals. As a result, for each signal sample, 24 × 2 × (*M* + 1) features will be extracted, where *M* is the number of chosen IMFs. In this paper, the first 5 IMFs will be selected for analysis, so the original feature vector of each signal will contain 288 features.

### 4.3. The Proposed 3-Stage Hybrid Feature Selection Framework

Filter models evaluate features from different perspectives, measure the possible characterization ability under a single measure, and can be effective for rapid feature ranking. However, due to different evaluation perspectives, the outcomes of various Filter models may be highly dissimilar. The same feature may be rejected as irrelevant in 1 model while accepted as sensitive in another. In order to provide pre-ranking results from various perspectives, this paper uses the FS, IG, and PCC methods in the first stage to evaluate features from the perspectives of distance, information, and relevance, respectively.

However, these evaluation results alone cannot eliminate redundant features. When feature a and feature b, for instance, both have high Fisher scores and a strong correlation, removing 1 of them won’t have an impact on the subsequent training process and the learning performance won’t be lost, but the FS model will keep both of them, leaving the feature set with redundant features. The same is true of PCC and IG models, but the evaluation results of PCC and IG methods can effectively exclude redundant features while being relative to select sensitive features, so these 3 sets of assessment findings can each provide complementary information to 1 another. Therefore, in order to reduce the number of redundant features and eliminate irrelevant features from the original feature set, as well as to minimize the dimensionality of the original set and maintain the capacity for classification, it is possible to combine the pre-ranking results from the 3 models. In addition, the sole use of the Filter model also ignores the potential gain in characterization ability that results from the combination of features. For instance, whereas features a and b individually have extremely low scores, combining them results in potentially very high scores, indicating that using features a and b together can yield significantly greater benefits for fault classification tasks. Filter models will, however, eliminate features a and b because they only calculate the scores of each feature individually without taking into account the combined impact between features, which plainly results in a waste of learning performance.

In order to address the aforementioned 2 shortcomings, a method is required to synthesize the assessment findings of the 3 Filter models. This is because the characterization ability of features can only be better compared when they are utilized in combination. In the weighting scheme proposed by ZHANG et al. [45], the accuracy of the classifier is used as a weight, and the pre-ranking results of the Filter model are combined to synthesize a new fusion evaluation result. The scheme first chooses the top *k* features in the pre-ranking result, then inputs a subset consisting of these *k* features into the classifier for training. The score for the corresponding feature is calculated as the product of the obtained classification accuracy and the feature’s ranking result, and the weighted score for the feature is calculated as the cumulative sum of multiple Filter models, as given in Equation (9).

(9) $$\{\begin{array}{l} Score_{i}=Ranking_{i}\times Accuracy_{i}\\ NewScore=\sum_{i=1}^{3}Score_{i} \end{array}$$

where *Score_i_* represents the score of the *i*th Filter method on the features, *i* denotes the FS, PCC and IG models, respectively, and *NewScore* represents the weighted score.

This scheme achieves impressive performance on single-label data but is not applicable to the case of multi-label data. This is because, in single-label learning, the labels are only predicted correctly or incorrectly. However, in the case of multi-label learning, it is possible that some of the predicted labels will match the real ones while others won’t. Therefore, it is also necessary to account for this partially correct scenario using a revised fitness function in this paper, as illustrated in Equation (10).

(10) $$Fit=\frac{(1+\beta^{2})\times SA\times AP}{\beta^{2}\times SA+AP}$$

where *SA* represents severe accuracy, and *AP* represents average accuracy. It is clear that the influence of *SA* on the fitness function dominates when *β* is less than 1, while AP dominates when *β* is greater than 1.

Additionally, the scheme depicted in Equation (9) simply merges the evaluation results of Filter models without realizing that the evaluation results of different Filter models do not contribute exactly to the classification task and overly idealizes the weight of *Score_i_* as equal. As a result, this paper applies integration techniques to combine the 3 pre-ranking outcomes. The weighted average method, which obtains the fusion results by performing a weighted average for a set of data, is the most common and straightforward integration technique. Therefore, this paper will combine the evaluation results of the 3 Filter models by assigning appropriate weights to *Score_i_* through the weighted average method on the basis of Equation (9), as indicated in Equation (11).

(11) $$\{\begin{array}{l} FusionScore=\sum_{i=1}^{3}\omega_{i}Score_{i}\\ \sum_{i=1}^{3}\omega_{i}=1 \end{array}$$

where *Score_i_* represents the score of the *i*th Filter method on the features, *ω_i_* ≥ 0, and *FusionScore* represents the weighted fusion score.

**Figure 4:**
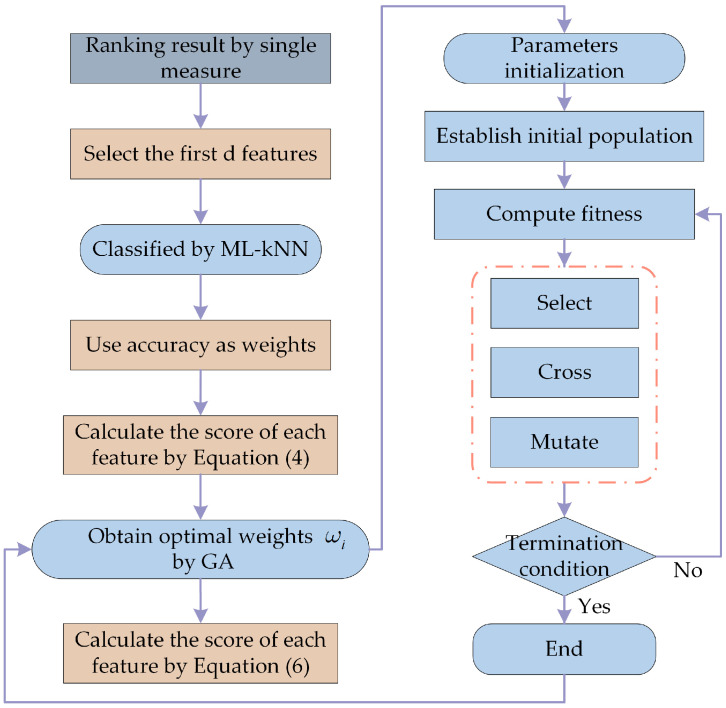
The flowchart of the proposed weighting scheme.

Obviously, there are an unlimited number of combinations of weights *ω_i_*, which will be solved by genetic algorithm in this paper. The weights *ω_i_* solved by generation are transformed into individuals in the initial randomized population by binary coding, and the optimal solution of weights *ω_i_* is obtained after several iterations of selection, crossover and variation. The first *k* features with the highest score in each individual are chosen as the subset and fed into the classifier. The fitness of each individual is calculated through Equation (10), and the optimal individual in each iteration is retained. The complete process of the weighting scheme is shown in Figure 4.

The weighting scheme determines the ranking order of the sensitivity of the candidate features, but it cannot directly locate the optimal subset from it. The Wrapper model has an unmatched advantage over the Filter model in terms of pinpointing an optimal subset, but it also has a high temporal complexity, particularly in the case of blind search. However, if there is a clear search direction, the time complexity of using the Wrapper model to find the optimal subset can be greatly reduced. Because the fusion ranking results are already ranked according to the strength of the feature representation, the Wrapper model’s necessary search direction is also identified. Therefore, finding the optimal subset of the range from the weighted re-ranking results of features just requires a few quick and effective search algorithms. In this paper, a mixture of 3 search strategies will be employed to enhance search speed and guarantee search quality. Briefly, The BS strategy is used to quickly eliminate a large number of irrelevant features and determine the approximate range of the optimal subset. The results of the BS strategy are then used as the initial subset, followed by the SFS and SBS strategies are used to search 1 by 1, and finally, the results of the 2 strategies are compared, and the 1 with the highest classification accuracy is retained, to achieve the precise location of the optimal subset. Naturally, the BS, SFS, and SBS strategies all take into account how features work together during the classification process.

**Figure 5:**
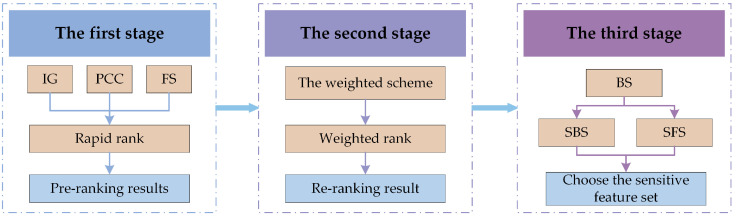
The flowchart of the proposed three-stage hybrid feature selection framework.

The features that are irrelevant and redundant to the classification objective will finally be removed from the original feature set through the 3-stage progression. In order to maintain or even increase the accuracy of fault classification while reducing the dimensionality of the feature set, the obtained feature subset will be the optimal 1 that considers both the characterization ability of the individual features and the characterization ability of the combined features. Figure 5 depicts the whole flowchart of the proposed three-stage hybrid feature selection framework, and the effectiveness of this framework will be tested in the following section.

## 5. Experiments and Analyses

### 5.1. Experimental Apparatus and Data Acquisition

**Figure 6:**
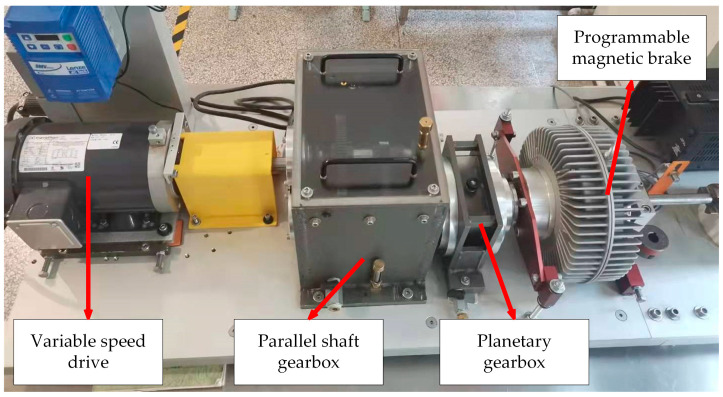
Wind turbine drive system fault simulation test bench.

The vibration signal samples used in this paper were obtained using a wind turbine drive system fault simulation test bench (WTDS) manufactured by Spectra Quest. According to Figure 6, the test bench is primarily made up of a variable speed drive, a planetary gearbox, a two-stage parallel shaft gearbox, and a programmed magnetic brake. Among them, the magnetic brake simulates the operating load by applying torque by modulating the input current. The motor will maintain a constant speed of 1000 r/min throughout the acquisition process for the test sample.

**Figure 7:**
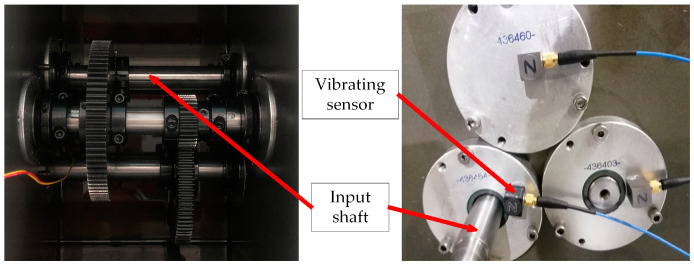
The installation position of the vibrating sensor.

As illustrated in Figure 7, the faulty parts were all mounted on the parallel shaft gearbox’s low-speed shaft, where they were detected by the vibrating sensor on the bearing end cover. The sampling frequency is adjusted to 20.48 kHz during acquisition, and the single fault or compound fault is introduced by swapping out several faulty parts. The collected fault categories include normal state, five single faults and three compound faults formed by coupling the single fault of gear and single fault of bearing. Table 1 displays the nine fault categories that were acquired overall. Each class intercepts 200 samples with 5000 points of segment length, and for each class, samples are completely randomly divided into training and testing sets in the ratio of 1:1. In total, there are 1800 samples for nine classes, 900 of which are the training data and the remaining 900 are the testing data.

### 5.2. Experimental Results and Analysis

By preprocessing the raw vibration signals and extracting the statistical features, each sample has 288 features, and the gearbox case, which contains nine state types, obtains an original high-dimensional set of 1800 × 288-dimensional. If all the original features are used as inputs to the classifier, the computational costs will be significant, and the classifier might not produce optimal results. Therefore, it is necessary to use the feature selection method to screen out the most beneficial feature subset for the fault diagnosis task. The characterization ability of these features is currently unclear, so first, a rapid pre-ranking of these 288 features is performed by three Filter models as described above, and the pre-ranking results are shown in columns 2–4 in Table 2. For instance, the feature numbered 1 is ranked ninth in the FS model, 33rd in the IG model, and 71st in the PCC model, while the feature numbered 288 is ranked 210th in the FS model, 203rd in the IG model, and 134th in the PCC model. This shows that different models produce different results for the same feature, so the evaluation outcomes of fault features from various evaluation viewpoints are all distinct. Therefore, multiple measurements should be combined to get evaluation findings that are more accurate and comprehensive.

These pre-ranking results of three Filter models are combined using a weighting scheme. The training set is utilized to measure the classification accuracy after a subset of features comprised of the top 20 ranked features from each method is fed into the ML-kNN classifier. The average accuracy was calculated independently after this procedure had been carried out ten times, and the resulting values were FS: 0.8967, IG: 0.9167, and PCC: 0.7956. The genetic algorithm was then used to find the weights *ω_i_* in Equation (11), with the population size set to 20, the maximum number of generations to 50, and the crossover rate and mutation rate of the genetic algorithm set to 0.8 and 0.1, respectively. Equation (10) was used to calculate fitness from the first 20 features of each individual. In this equation, *β* was set to 0.7, and the weights *ω_i_* of the three sets of *Score* were ultimately determined to be 0.7261, 0.2325 and 0.0414, respectively. Finally, the *FusionScore* is synthesized by Equation (11):

For the feature numbered 1, its *FusionScore* is calculated as follows:

0.7261 × 0.8967 × 9 + 0.2325 × 0.9167 × 33 + 0.0414 × 0.7956 × 71 = 15.2318

For the feature numbered 288, its *FusionScore* is calculated as follows:

0.7261 × 0.8967 × 210 + 0.2325 × 0.9167 × 203 + 0.0414 × 0.7956 × 134 = 184.4093

According to their *FusionScore*, each feature in the original feature set is ranked in descending order, as seen in the final column of Table 2. At this time, the feature numbered 1 is ranked 10th in the weighting scheme ranking, whereas the feature numbered 288 is placed 203rd.

In this case, the higher the ranking of the features in the fusion ranking result, the better the performance in the fault classification task. The BS strategy was first used to expedite the search process by dividing the original feature set into a left subset and a right subset and measuring the recognition accuracy of the two subsets separately in order to precisely determine the features contained in the optimal subset. For the first iteration, the left subset’s subset accuracy is 94.11%, while the right subset’s subset accuracy is substantially lower at just 60.89%; hence, the left subset is chosen for future investigation.

**Figure 8:**
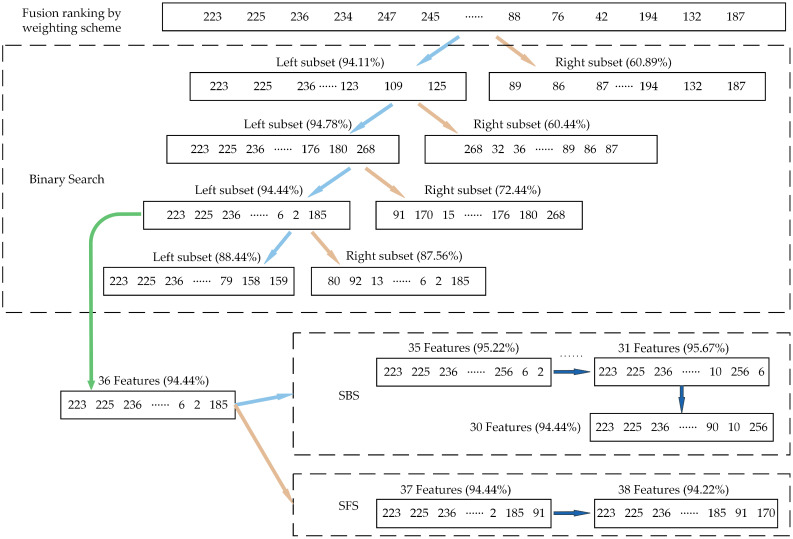
The visual presentation of the iterative search process for Case 1.

This procedure was repeated until the subset accuracy of the left subset and right subset fell below the threshold (94%), as seen in Figure 8. After three iterations, the original feature set’s 288 features had been reduced to 36 features. Then, using the SBS and SFS strategies, respectively, these 36 features were used as the initial subset. While the SFS strategy stopped after 36 features had been searched and had an accuracy of 94.44%, the SBS method stopped after 31 features had been searched and had an accuracy of 95.67%. In contrast, the SBS strategy produced search results with higher accuracy; hence, its search results were regarded as the optimal subset, and the feature selection process was finished; the original 1800 × 288-dimensional feature set was reduced to 1800 × 31-dimensional.

**Figure 9:**
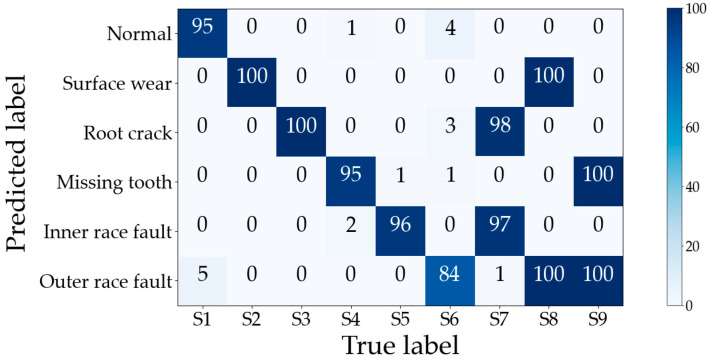
Gearbox compound fault dataset classification confusion matrix for Case 1.

The optimal subset consisting of these 31 features in the training set is input into the ML-kNN classifier, and the testing set validates the performance of feature selection; the predicted labels are shown in Figure 9. The horizontal axis indicates the number of labels predicted to correspond to a certain fault type in the current sample, and it can be found that both single and compound faults are effectively identified. The compound faults are successfully decoupled into two single faults, such as the labels corresponding to two single faults, S4 and S6, which are predicted according to the compound fault sample S9. The labels corresponding to S2 and S6 are predicted to the compound fault sample S8.

The preceding analysis revealed that partial correctness should also be taken into account in multi-label classification. Therefore, in addition to subset accuracy (SA) and average precision (AP), the following four indicators were chosen as assessment indicators to properly assess the performance of the proposed method: Hamming loss (HL), ranking loss (RL), one-error (OE), and coverage (Cov) [18], and these six indicators are calculated as follows:

(12) $$SA(h)=\frac{1}{p}\sum_{i=1}^{p}〚h(x_{i})=Y_{i}〛$$

(13) $$AP(h)=\frac{1}{p}\sum_{i=1}^{p}\frac{1}{\left|Y_{i}\right|}\sum_{y\in Y_{i}}\frac{\left|\left\{y^{\prime }|rank_{h}(x_{i},y^{\prime })\leq rank_{h}(x_{i},y),y^{\prime }\in Y_{i}\right\}\right|}{rank_{h}(x_{i},y)}$$

(14) $$HL(h)=\frac{1}{p}\sum_{i=1}^{p}\frac{1}{q}\left|h(x_{i})△Y_{i}\right|$$

(15) $$OE(h)=\frac{1}{p}\sum_{i=1}^{p}〚\left[\arg\max_{y\in L}h(x_{i},y)\right]\notin Y_{i}〛$$

(16) $$RL(h)=\frac{1}{p}\sum_{i=1}^{p}\frac{1}{\left|Y_{i}\right|\left|{\bar{Y}}_{i}\right|}\sum_{y\in Y_{i}}\left\{(y^{\prime },y^{\prime \prime })|h(x_{i},y^{\prime })\leq h(x_{i},y^{\prime \prime })\right\}$$

(17) $$Cov(h)=\frac{1}{p}\sum_{i=1}^{p}\max_{y\in Y_{i}}rank_{h}(x_{i},y)-1$$

where ⟦ ⟧ represents that the condition returns 1 if true and 0 otherwise, *h*( ) represents the trained classifier, *Y_i_* represents the label vector corresponding to the test sample *x_i_*, △ represents the symmetric difference between two sets, and |*A*| represents the base of set *A*.

It is straightforward to see from the expressions that the larger the value of subset accuracy and average precision, the better the performance, while the smaller the value of Hamming loss, ranking loss, one-error, and coverage, the better the performance. We will measure the performance of the proposed method by comparing these six indicators in the testing set. First, comparing the diagnostic performance of the feature set before and after feature selection, the results are listed in Table 3. From another standpoint, the computational cost can also be used to measure the performance of the classification, so this paper also compares the average computational cost of the classifier for training and testing procedures (in seconds).

Table 3 shows that the optimal subset outperforms the original set in each of the six indicators, and the computational cost is also a bit lower. Since the definition of the subset accuracy is extremely strict, the original feature set performs much worse than the optimal subset in terms of subset accuracy, lagging by 10.00%, although its average accuracy only lags by 0.76%. This shows that the proposed feature selection and fault decoupling framework may be utilized to diagnose gearbox compound faults in an efficient manner, improving classification performance while requiring fewer features.

### 5.3. Comparison with Other Methods

**Figure 10:**
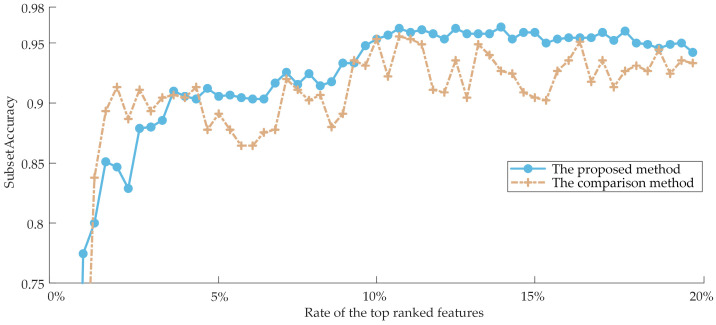
The comparison result between the proposed method and the previous method.

To demonstrate the superiority of the proposed method, we used the weighting scheme proposed by ZHANG et al. [45] as a comparison to synthesize the ranking results of three Filter models by Equation (9) only, compared the classification performance of features in the two sets of weighted ranking results, and the variation curve of the subset Tab;ccuracy with the number of features was plotted, as shown in Figure 10. In Figure 10, the horizontal axis indicates the percentage of the top-ranked features in the fusion ranking results. Generally speaking, the classification performance caused by selecting the top *k* features will be higher than selecting the top *k* − 1 features and lower than selecting the top *k* + 1 features. However, the classification performance is not simply superimposed due to the combined effect among the features, so the curve exhibits an oscillation phenomenon. But the overall performance still gradually improves with the addition of more features, so there is no significant shift in the general trend.

Analyzing Figure 10, it is intuitively clear that as the number of features rises, both methods’ performance in terms of subset accuracy steadily improves and finally stabilizes. However, the proposed weighting scheme can obtain higher subset accuracy, and the oscillation phenomenon is weaker than the comparison method, as shown by the fact that the proposed method is more reasonable and can assign appropriate weights according to their contribution degree of various Filter models for the better synthesis of the re-ranking results.

**Figure 11:**
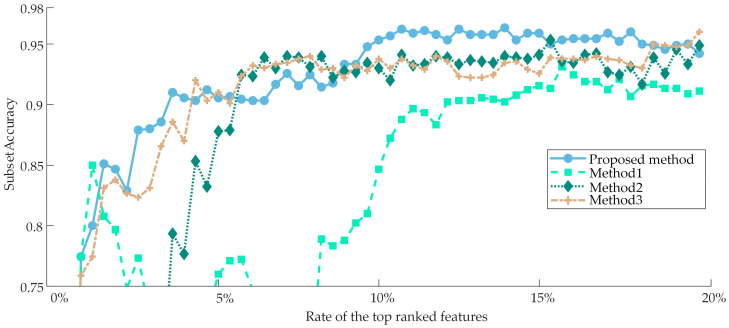
The comparison result between the proposed method and other methods for Case 1 in terms of subset accuracy.

The proposed method is compared with three representative classical methods: the PPT + MI [46], LP + CHI [47], and PPT + Relief-F [37], described as Method 1, Method 2, and Method 3, respectively. In Method 1, multi-label data was initially transformed to single-label data using the pruned problem transformation (PPT), after which the optimal features in the converted feature set were chosen based on mutual information. And in Method 3, which is similar to Method 1, the best features are selected by using Relief in the converted feature set. In Method 2, the multi-label data is transformed to single-label data by LP as the proposed method, but the optimal features are selected only by CHI in the converted feature set. The variation curves of subset accuracy with the number of features are plotted as shown in Figure 11 for a more understandable comparison since these three algorithms cannot accurately obtain the optimal subset automatically and only rely on methods like setting thresholds to end the selection process. As can be observed by the fact that Method 2 requires the fewest features to achieve its highest subset accuracy, but the difference between Method 2 and Method 3 is relatively slight, and the curves of both are almost always in the intersection. However, the highest subset accuracy achieved by both of them is much lower than that of the proposed method, and they are quickly caught up and surpassed by the proposed method, which consistently outperforms the other three methods after using 10% of the features. Method 1, on the other hand, has the best performance for a short time in the beginning, and then its performance is consistently lower than the other methods; nevertheless, when the number of features rises, Method 1’s performance eventually catches up to Method 2 and Method 3.

Table 4 lists the four methods’ top performances, with the best outcomes in each column denoted by bold type. Since Method 1 and Method 3 evaluate the features from the distance measure by CHI and Relief-F, respectively, the selected features are the most beneficial to the classification task, and the subset accuracy is improved the quickest. However, due to the existence of redundant features, the maximum accuracy they can achieve is lower than that of the proposed method. While Method 2 evaluates the features from the information measured by MI, so its improvement speed of subset accuracy is relatively slowest, and it is also unable to avoid redundant features. The proposed method takes into account all three measures and is able to eliminate the redundant and irrelevant features in the subset to the maximum extent. The other three methods, which required the usage of more features, only achieved subset accuracy of 93.11%, 95.33%, and 96.00%, whereas the proposed method produced a subset accuracy of 96.22%. In all six indicators, Method 1 and Method 2 fared worse than the other two methods and failed to win any of them. Method 3, on the other hand, achieved a Hamming loss that was extremely consistent with the proposed method and performed quite similarly in terms of diagnostics. Several other indicators also won and lost against each other, with the differences being minor. However, Method 3 chooses roughly twice as many optimal features as the proposed method, which means the classifier must be trained for a longer period. This indicates that the proposed method can obtain higher diagnostic performance with fewer features by removing as many irrelevant and redundant features from the original feature set as is practicable.

### 5.4. Added Experiments

**Figure 12:**
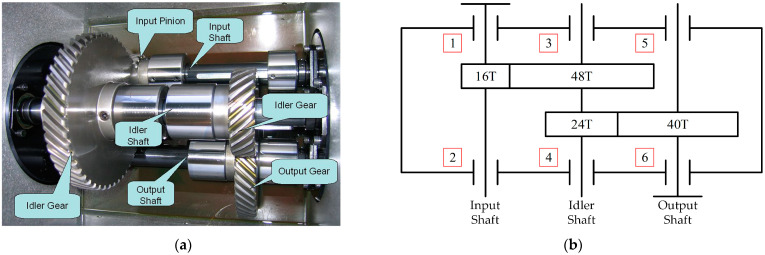
A pictorial illustration of the experimental gearbox. (**a**) Gearbox interior. (**b**) Schematic diagram of gearbox structure.

To validate the generality of the proposed method, the proposed method was validated using the PHM09 dataset, which was obtained from a challenge organized by the Prognostics and Health Management Association in 2009 [48], using a general-purpose industrial gearbox measured as shown in Figure 12, including three shafts, two pairs of helical gears, and six bearings. The dataset was collected for failures at 30, 35, 40, 45, and 50 Hz speeds under high and low load scenarios, respectively, which did not include repeated trials.

Six fault types collected at 2400 r/min and low load were used, with a sampling frequency of 66.67 kHz. Table 5 displays the individual fault kinds, and the faults are highlighted in bold throughout the table. For each fault state, 80 samples were intercepted with 5000 points of segment length for each one and split into training and testing sets in a 1:1 ratio. We refer to the dataset obtained through WTDS as Case 1 and the dataset obtained using PHM09 as Case 2.

For Case 2, a 480 × 288-dimensional original high-dimensional feature set was generated, similar to how Case 1’s features were extracted. The ability of each feature in the feature set generated for a given dataset to describe the operating state of the gearbox is very different for different datasets, so the 420 features are still evaluated by three filter models first and pre-ranked in descending order according to the evaluation results. The pre-ranking results of the features in each method are displayed in columns two to four of Table 6. The feature subset consisting of the top 10 features in each method was selected, and input into the ML-kNN classifier, and the classification accuracy was estimated by the training set; the results obtained were FS: 0.9583, IG: 1.0000, and PCC: 0.7375. The genetic algorithm was then used to find the weights *ω_i_* in Equation (11), with the population size set to 10, the maximum number of generations 50, and the crossover rate and mutation rate of the genetic algorithm set to 0.8 and 0.1, respectively. Equation (10) was used to calculate fitness from the first 10 features of each individual. In this equation, *β* was set to 0.7, and the weights *ω_i_* of the three sets of *Score* were ultimately determined to be 0.6740, 0.3064, and 0.0196, respectively. Equation (11) was then used to synthesize the *FusionScore*. According to their *FusionScore*, each feature in the feature set is ordered in the last column of Table 6 in ascending order.

**Figure 13:**
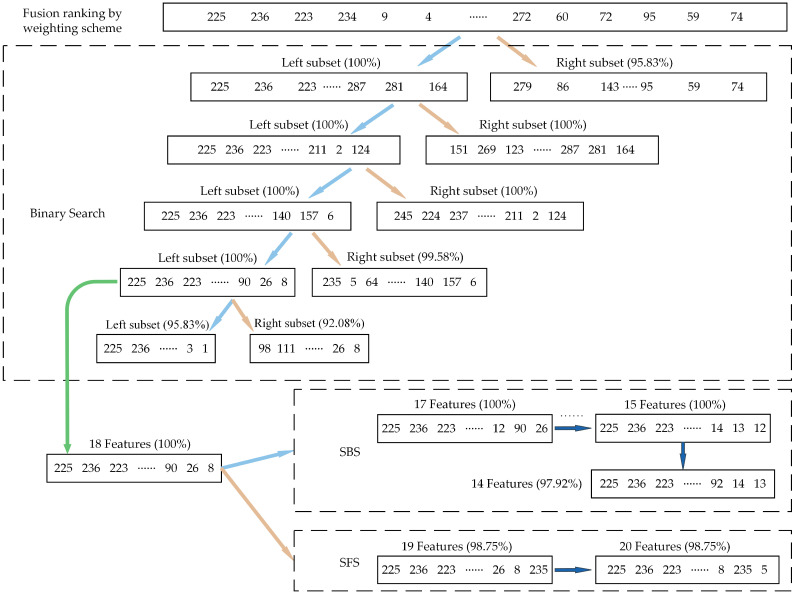
The visual presentation of the iterative search process for Case 2.

The search process was then sped up by the BS strategy at the iterative search stage in order to discover the precise range of the optimal subset, as shown in Figure 13. The number of features in the original feature set was reduced from 288 to 18 after 4 iterations. The SBS and SFS strategies were then used to iterate the search once more using these 18 features as the initial subset. The SBS strategy stops at 15 features, at which time the subset accuracy is 100.00%, while the SFS strategy stops at 18 features, at which time the subset accuracy is also 100.00%. In contrast, the SBS strategy’s search results include fewer features; therefore, this search result is considered to be the optimal subset, and the feature selection process is completed; the original 480 × 288-dimensional feature set has been reduced to 480 × 15-dimensional.

**Figure 14:**
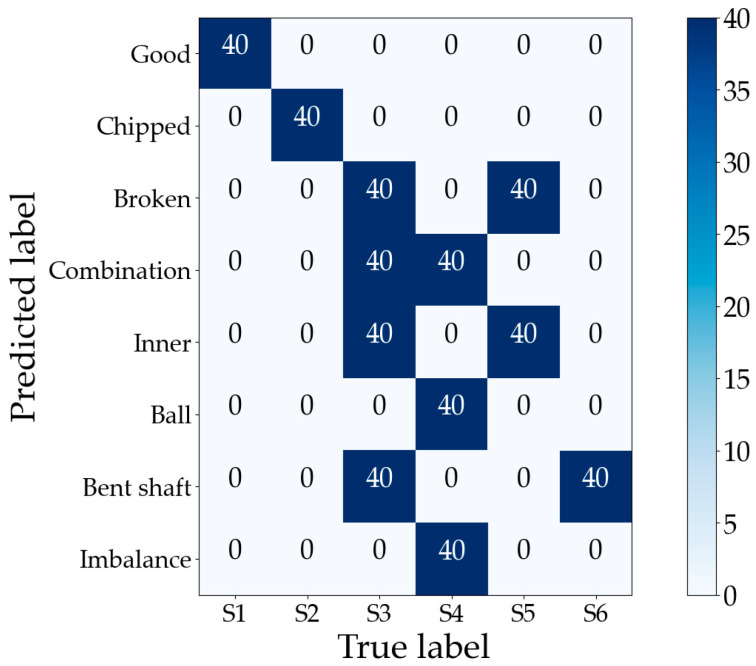
Gearbox compound fault dataset classification confusion matrix for Case 2.

The training set’s optimal subset is fed into ML-kNN for training, and the trained ML-kNN is used to diagnose the testing set with the predicted labels as shown in Figure 14, the horizontal axis indicating the number of labels corresponding to the current sample that is predicted to be a certain fault type. The samples in the PHM09 dataset are coupled to more fault states than in Case 1—four parts of S3 generate faults simultaneously, and three parts of S4 generate faults simultaneously—but the proposed method can still fully decouple them into multiple single faults which are coupled to form that compound fault state.

**Figure 15:**
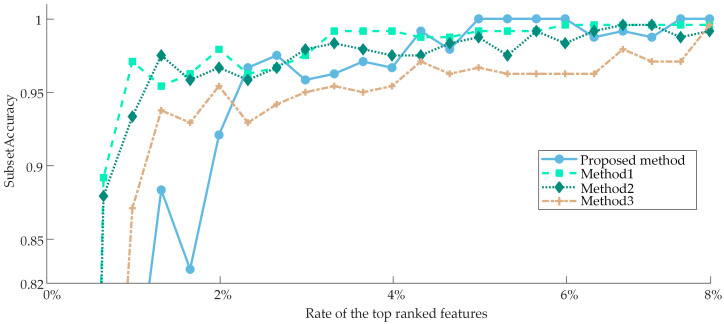
The comparison result between the proposed method and other methods for Case 2 in terms of subset accuracy.

The original high-dimensional feature set achieved a subset accuracy of 98.75%, attributable to the PHM09 dataset’s exceptionally ideal acquisition conditions, while the optimal subset achieved a subset accuracy of 100%. Figure 15 depicts the variation curves of the subset accuracy of the four methods with the number of features to make visual observation easier. The proposed method still performs the best, despite the fact that the margin between the four methods is significantly narrower than it was in Case 1. Only the proposed method reaches 100% subset accuracy when only the first 6% of features are employed, despite the fact that the subset accuracy of all four methods can rise as the number of features increases. Method 1 and Method 2 perform similarly and slightly worse than the proposed method, while Method 3 performs the worst, with a subset accuracy of about 96%.

In Case 2, the proposed method automatically determines an optimal subset with 15 features. The diagnostic performance of three different methods is compared when employing the top 15 features from their ranking results in order to more clearly illustrate the diagnostic benefits of the proposed feature selection and fault decoupling method. Table 7 summarizes the subset accuracy, average precision and Hamming loss of each method. It is clear that in Case 2, the proposed method still has the best performance, as it outperforms the other models in all evaluation metrics. Method 3 performs worse than Method 1, which is the opposite of Case 1, indicating that their performance is less stable than the proposed method in different source datasets.

Both Table 4 and Table 7 demonstrate that the proposed method has stable performance and is effective in feature selection and fault decoupling in both Case 1 and Case 2. The method can guarantee better accuracy while using the fewest possible feature dimensions. Therefore, we can conclude that the method performs better in terms of test accuracy than the three methods that were compared.

## 6. Conclusions and Future Works

This paper proposes an effective feature selection and fault decoupling framework in the field of intelligent fault diagnosis, which can achieve the highest diagnostic accuracy. The proposed method’s experimental findings on two gearbox datasets demonstrate that it can diagnose more accurately while using fewer features than previous methods. It also has the advantages of simple computation and high accuracy, which increases the usefulness and efficacy of intelligent diagnosis models. From this research, the following findings can be made:

- In order to describe the operating condition of the gearbox as comprehensively as possible, we propose to construct multi-domain feature sets. The Teager energy operator and the EMD algorithm are utilized in the proposed feature extraction stage to filter out disturbing components from the signal and highlight the fault feature information. Each vibration signal is thus given a high-dimensional feature vector that contains irrelevant and redundant features, and it is important to adaptively screen the most sensitive features using feature selection techniques;
- The original multi-label problem will be taken again into account to achieve multi-label classification by the AA method once the feature selection for the original feature set has been completed by employing the PT method. Consequently, the feature selection and fault decoupling framework proposed in this paper is more adaptable;
- The original high-dimensional feature set contained redundant and irrelevant features, which may have increased training time and decreased recognition accuracy. The ML-kNN is utilized as the classifier in the proposed feature selection method, and three Filter models are initially employed to pre-rank the features. To achieve a reranking result that takes into account feature irrelevance, redundancy, and inter-feature interaction factors, the pre-ranking results are fused using a weighting scheme. In order to optimize the subset for the classifier and attain the highest diagnostic accuracy, a search stage that could adaptively discover the optimal subset is constructed based on three heuristic search strategies;
- The proposed method does not entail complicated mapping, which is an intuitive and straightforward process. Therefore, this method has a good physical interpretation and helps to reveal the connection between faults and their related features, offering a new solution for diagnosing compound defects in gearboxes.

The most significant advantage of the proposed method over other methods is that better fault diagnosis performance can be achieved, but at the same time, the computational overhead in the feature selection stage is relatively high. Therefore it should also be considered in subsequent studies how to reduce computational costs while maintaining diagnostic performance. In addition, in this work, we transformed the multi-label data by label powerset, which may perform less well in datasets with imbalanced samples or a lot of label space. Therefore, more suitable processing methods need to be tried, which is the direction that the authors would make further efforts.

## Figures and Tables

**Table 1:** Testing data description.

Fault Types	S1	S2	S3	S4	S5	S6	S7	S8	S9
Normal	√								
Gear surface wear		√						√	
Gear root crack			√				√		
Gear missing tooth				√					√
Bearing inner race fault					√		√		
Bearing outer race fault						√		√	√

From the table, √ indicates the fault type in the sample corresponding to each column.

**Table 2:** Ranking results for features of different methods for Case 1.

Order	FS	IG	PCC	Weighting Scheme
1	223	146	240	223
2	225	159	229	225
3	236	172	251	236
4	234	185	262	234
5	247	236	273	247
6	106	223	172	245
7	245	234	146	235
8	107	198	185	226
9	1	211	159	107
10	226	225	228	1
…	…	…	…	…
285	34	141	261	42
286	99	143	139	194
287	194	121	239	132
288	88	128	106	187

**Table 3:** Performance comparison with the origin feature set and the optimal subset.

	Computational Cost (s)	SA	AP	HL	RL	Cov	OE
Origin feature set	0.971	0.8622	0.9765	0.0278	0.0114	0.3878	0.0444
Optimal subset	0.469	0.9622	0.9841	0.0113	0.0084	0.3689	0.0300

**Table 4:** Performance comparison with other frameworks for Case 1.

Method	Number of Optimal Features	Computational Cost (s)	SA	AP	HL	RL	Cov	OE
The proposed method	**31**	**0.469**	**0.9622**	0.9841	**0.0113**	**0.0084**	0.3689	0.0300
Method 1	45	0.489	0.9311	0.9730	0.0187	0.0124	0.3956	0.0500
Method 2	44	0.489	0.9533	0.9765	0.0150	0.0096	0.3811	0.0467
Method 3	57	0.517	0.9600	**0.9843**	**0.0113**	0.0088	**0.3678**	**0.0278**

From the table, the bold indicates the best performance in each column.

**Table 5:** Testing data description.

Case	Gear				Bearing						Shaft	
	16T	48T	24T	40T	1	2	3	4	5	6	Input	Output
S1	Good	Good	Good	Good	Good	Good	Good	Good	Good	Good	Good	Good
S2	Good	Good	**Chipped**	Good	Good	Good	Good	Good	Good	Good	Good	Good
S3	Good	Good	**Broken**	Good	Good	Good	Good	**Combination**	**Inner**	Good	**Bent shaft**	Good
S4	Good	Good	Good	Good	Good	Good	Good	**Combination**	**Ball**	Good	**Imbalance**	Good
S5	Good	Good	**Broken**	Good	Good	Good	Good	Good	**Inner**	Good	Good	Good
S6	Good	Good	Good	Good	Good	Good	Good	Good	Good	Good	**Bent shaft**	Good

From the table, the bold indicates the specific fault type.

**Table 6:** Ranking results for features of different methods for Case 2.

Order	FS	IG	PCC	Weighting Scheme
1	225	80	229	225
2	236	223	251	236
3	223	82	240	223
4	9	88	228	234
5	234	5	254	9
6	11	6	96	4
7	1	7	262	11
8	88	14	51	3
9	5	91	253	1
10	80	10	232	81
…	…	…	…	…
285	33	63	283	72
286	34	142	31	95
287	59	144	57	59
288	74	143	139	74

**Table 7:** Performance comparison with other frameworks for Case 2.

Method	SA	AP	HL
The proposed method	1.0000	1.0000	0.0000
Method 1	0.9917	1.0000	0.0021
Method 2	0.9875	1.0000	0.0031
Method 3	0.9667	0.9861	0.0078

## Data Availability

The data presented in this study are available upon request from the corresponding author. The data are not publicly available due to laboratory regulations.

## Appendix A

The equation of statistical features adopted in this work is shown in Table A1.

**Table A1:** Statistical feature of the time and frequency domains.

Time-Domain Feature		Frequency-Domain Feature	
$T_{1}=\frac{\sum_{n=1}^{N}x(n)}{N}$	$T_{7}=\frac{\sum_{n=1}^{N}{\left(x(n)-T_{1}\right)}^{4}}{(N-1)T_{2}{}^{4}}$	$F_{1}=\frac{\sum_{k=1}^{K}s(k)}{K}$	$F_{8}=\sqrt{\frac{\sum_{k=1}^{K}f_{k}{}^{4}s(k)}{\sum_{k=1}^{K}f_{k}{}^{2}s(k)}}$
$T_{2}=\sqrt{\frac{\sum_{n=1}^{N}{\left(x(n)-T_{1}\right)}^{2}}{N-1}}$	$T_{8}=\frac{T_{5}}{T_{4}}$	$F_{2}=\frac{\sum_{k=1}^{K}{\left(s(k)-F_{1}\right)}^{2}}{K-1}$	$F_{9}=\frac{\sum_{k=1}^{K}f_{k}{}^{2}s(k)}{\sqrt{\sum_{k=1}^{K}s(k)\sum_{k=1}^{K}f_{k}{}^{2}s(k)}}$
$T_{3}={\left(\frac{\sum_{n=1}^{N}\sqrt{\left|x(n)\right|}}{N}\right)}^{2}$	$T_{9}=\frac{T_{5}}{T_{3}}$	$F_{3}=\frac{\sum_{k=1}^{K}{\left(s(k)-F_{1}\right)}^{3}}{K{\left(\sqrt{F_{2}}\right)}^{3}}$	$F_{10}=\frac{F_{6}}{F_{5}}$
$T_{4}=\sqrt{\frac{\sum_{n=1}^{N}{\left(x(n)\right)}^{2}}{N}}$	$T_{10}=\frac{T_{4}}{\frac{1}{N}\sum_{n=1}^{N}\left|x(n)\right|}$	$F_{4}=\frac{\sum_{k=1}^{K}{\left(s(k)-F_{1}\right)}^{4}}{KF_{2}{}^{2}}$	$F_{11}=\frac{\sum_{k=1}^{K}{\left(f_{k}-F_{5}\right)}^{3}s(k)}{KF_{6}{}^{3}}$
$T_{5}=\max\left|x(n)\right|$	$T_{11}=\frac{T_{5}}{\frac{1}{N}\sum_{n=1}^{N}\left|x(n)\right|}$	$F_{5}=\frac{\sum_{k=1}^{K}f_{k}s(k)}{\sum_{k=1}^{K}s(k)}$	$F_{12}=\frac{\sum_{k=1}^{K}{\left(f_{k}-F_{5}\right)}^{4}s(k)}{KF_{6}{}^{4}}$
$T_{6}=\frac{\sum_{n=1}^{N}{\left(x(n)-T_{1}\right)}^{3}}{(N-1)T_{2}{}^{3}}$		$F_{6}=\sqrt{\frac{\sum_{k=1}^{K}{\left(f_{k}-F_{5}\right)}^{2}s(k)}{K}}$	$F_{13}=\frac{\sum_{k=1}^{K}\sqrt{\left(f_{k}-F_{5}\right)}s(k)}{K\sqrt{F_{6}}}$
		$F_{7}=\sqrt{\frac{\sum_{k=1}^{K}f_{k}{}^{2}s(k)}{\sum_{k=1}^{K}s(k)}}$	

From the table, *x*(*n*) is a signal series for *n*= 1, 2, …, *N*, *N* is the number of data points, *s*(*k*) is a spectrum for *k*= 1, 2, …, *K*, *K* is the number of spectrum lines, and *f_k_* is the frequency value of the *k*th spectrum line, *f*^ˆ^(*λ_l_*) is an eigenvector for *λ_l_*, *λ_l_* is the eigenvalue of the *l*th degree, *l* = 1, 2, …, *N*.
